# Evaluation on the Microstructure and Durability of High-Strength Concrete Containing Electric Arc Furnace Oxidizing Slag

**DOI:** 10.3390/ma14051304

**Published:** 2021-03-09

**Authors:** Tian-Feng Yuan, Se-Hee Hong, Jin-Seok Choi, Young-Soo Yoon

**Affiliations:** School of Civil, Environmental and Architectural Engineering, Korea University, 125 Anam-ro, Seongbuk-gu, Seoul 02841, Korea; yuantianfeng@korea.ac.kr (T.-F.Y.); bestshhong@korea.ac.kr (S.-H.H.); radiance@korea.ac.kr (J.-S.C.)

**Keywords:** electric arc furnace oxidizing slag, ground granulated blast furnace slag, cementitious material, microstructure, shrinkage properties, freezing and thawing resistance

## Abstract

The application of electric arc furnace oxidizing slag (EAS) in high strength concrete (HSC) as the cementitious material is investigated in this study. The microstructure and mechanical properties of HSC with four different replacement ratios of EAS were evaluated and HSC with two replacement ratios of ground granulated blast furnace slag (GBS) was used for performance comparison. The results show that the HSC with EAS replacement ratios smaller than 15% undergo similar hydration processes and result in a similar final product when compared with those of NC-NN. Increases in EAS replacement ratio cause a reduction in Ca(OH)_2_ content; this, in turn, leads to an increase in porosity and a reduction in compressive strength. In terms of shrinkage behavior under free conditions, mixtures with increasing replacement ratios of cementitious materials saw increasing shrinkage, with the HSC containing EAS being similar to the other specimens. The mixtures containing EAS saw a quite gradual decrease in their freezing and thawing resistance properties as the number of freeze–thaw cycles they underwent increased. However, the efficacy of HSC with less than 15% of EAS is similar to GBS; hence, EAS could replace cement in concrete for certain applications, which would lead to more environmental benefits.

## 1. Introduction

Electric arc furnace oxidizing slag (EAS) is generated by the iron and steel industry after an oxidizing process. Over 1600 million tons are produced annually worldwide [1,2]. To achieve sustainable development, we must recycle industrial waste; for this purpose, EAS is generally used as a raw material for bricks, ceramics, road fillers, and, in recent research, as an aggregate in concrete [1,3,4,5]. However, it is very rare to make concrete with EAS as the cementitious material. It is important to look for alternate ways to reuse EAS so as to contribute to sustainable development.

Several researchers [6,7,8,9] have been investigating the characteristics of EAS with a view to using it as a replacement material in concrete. The major chemical composition of EAS is SiO_2_, CaO, T-Fe* (total Fe_2_O_3_ and FeO), MgO, and Al_2_O_3_, the main mineral phases are C3S, C2S, RO phase, and *f*-CaO. The C3S and C2S are major components in the hydration reaction, while the RO phase is relatively stable, being crystallized and inert. EAS can be considered as a cementitious material due to its hydration properties being similar to those of cement, although the hydration rate is lower than that [10]. There is a lot of research where the possibility of EAS being used in concrete has been demonstrated. However, using EAS has led to negative properties in the concrete. Concrete with EAS has increased workability, but it has decreased hydration property in the matrix and decreased compressive strength [8,11,12]. In terms of durability, using EAS leads to reduced resistance to sulfate attack, chloride ion, and carbonation [13,14]. This is because the number of particles in EAS has poor cementitious properties and the interface between the particles of EAS and C2S hydrate gels deteriorates in the hardened matrix [13,14,15]. However, nearly all of the above research was focus on EAS applied as aggregate in concrete. There has hardly been any research on concrete made with EAS as the cementitious material; particularly, there was not any research that considered the EAS as the cementitious materials used in HSC (compressive strength over 60 MPa at 28 days).

Therefore, this study investigates the application of EAS in high strength concrete (HSC) as the cementitious material. Firstly, its microstructure properties were investigated by XRD analysis as well as porosity distribution, thermogravimetry/derivative thermogravimetry (TG/DTG) measurement. Then, the compressive properties of mixtures at the ages of 3, 7, 28, 56, and 90 days were evaluated, and an equation for predicting the compressive strength development in HSC with age was suggested. Thereafter, the shrinkage behavior (autogenous shrinkage and drying shrinkage) under free conditions as well as rapid freezing and thawing resistance were investigated. Therefore, this study is designed to contribute basic data on high-strength concrete containing electric arc furnace oxidizing slag.

## 2. Mixture Proportions and Materials

Table 1 details the mixture proportions used in this study. Following the previous study [16], three different water to binder ratios (w/b of 27.5%, 30.0%, 32.5%), and four different cement replacement ratio by EAS (0%, 10%, 15%, 20%) were used. It was found that mixtures with w/b of 32.5% have clearly the best mechanical properties; thus, only this was used. Thus, the w/b and fine aggregate ratios (s/a) were held constant at 32.5% and 40.3%, respectively. Type I Portland cement (Sampyo, Seoul, produced by the Republic of Korea) was used, which corresponds to ASTM C150 [17]. Electric arc furnace oxidizing slag (EAS) with a specific surface area of 4893 cm^2^/g and density of 3.96 g/cm^3^ and ground granulated blast furnace slag (GBS) with a specific surface area of 4250 cm^2^/g and density of 2.90 g/cm^3^ were adopted. The aging process (>4 months) was adopted to obtain stable EAS powder, in which the EAS is left in the air for *f*-CaO to get in touch with moisture from the air in order to change into Ca(OH)_2_. By adopting the treatments, the content of *f*-CaO is 0.8% in the EAS. Then the ball mill work was adopted to get the target specific surface area of EAS powder. The chemical and physical properties of the binder materials are listed in Table 2, these were determined by an X-ray fluorescence spectrometer (EZS003XNV, Korea Conformity Laboratories, Seoul, produced by the Republic of Korea). Compared to the chemical properties found in cement and GBS, the SiO_2_ and CaO contents in the EAS are significantly lower, that is, there is less calcium silicate. However, the T-Fe* (Fe_2_O_3_, FeO) content in the EAS is obviously higher than in other cementitious materials.

Crushed aggregate (limestone) was used for the fine aggregate (sand), and coarse aggregate (CA) with a maximum size of 20 mm was adopted. A poly-carboxylic acid-based air-entraining and water-reducing admixture (AEWR, Dongnamad, Seoul, produced by the Republic of Korea) was incorporated to achieve the required workability (180 ± 25 mm). All test specimens were cured in a room with a steady temperature (20 ± 1 °C) and humidity (65 ± 5%) until its designated test day.

## 3. Test Methods and Setup

### 3.1. Hydration Properties

The crystalline phases of cementitious materials (cement, EAS, BSF) and hydration products of HSC mixtures at an age of 28 days was determined by X-ray powder diffraction (XRD) analysis. 40 kV, 200 mA Cu Kα radiation and 0.01° at 0.3 s per step were adopted over a 2θ range of 10° to 60°. The Ca(OH)_2_ contents of the mixtures were calculated based on the thermogravimetry/derivative thermogravimetry (TG/DTG) curve at the age of 28 days. This was measured by a thermogravimetric analyzer in a throughflow nitrogen atmosphere, with a heating rate of 20 °C/min from 30 to 1300 °C. Furthermore, the porosity and pore size distribution were determined by mercury intrusion porosimetry (MIP), which is generally applied in the evaluation of pore characteristics for cementitious composites.

### 3.2. Compressive Strength Test

The compressive properties of mixtures at the ages of 3, 7, 28, 56, and 90 days were evaluated according to ASTM C39 [18]. A total of fifteen cylindrical specimens (diameter of 100 mm and height of 200 mm) for each mixture were fabricated for use in the unconfined compressive strength test. A uniaxial load was imposed using a displacement control rate of 0.1 mm/min by a UTM (universal testing machine, Minneapolis, MN, USA) with a maximum capacity of 250 t.

### 3.3. Free Shrinkage Test

**Figure 1:**
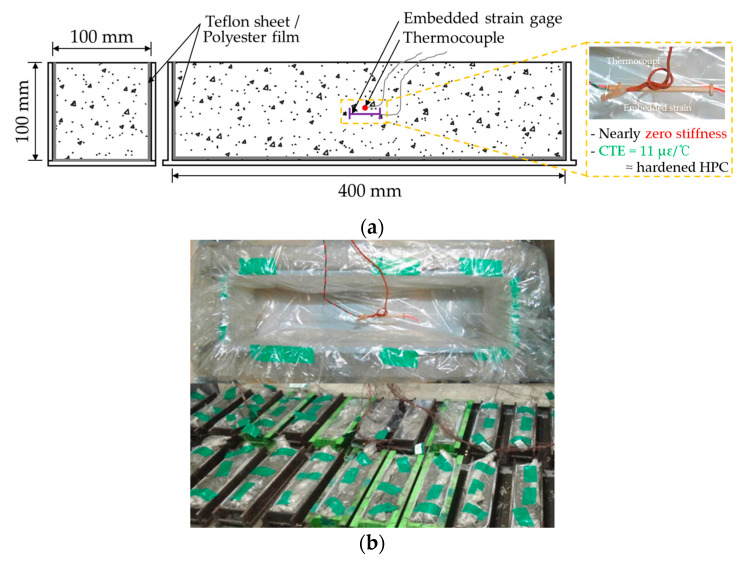
Shrinkage test under free condition: (**a**) conceptual figure of setup; (**b**) details of specimen after casting concrete.

Figure 1 shows the shrinkage test setup under free conditions (autogenous shrinkage and drying shrinkage). A total of three 100 × 100 × 400 mm prismatic specimens of each mixture were prepared according to the Japan Concrete Institute [19]. In order to eliminate the frictional force between the prismatic specimens and mold, a Teflon sheet and polyester film were used. To precisely evaluate the shrinkage from a very early age, and embedded strain gage which has nearly zero stiffness and a very similar coefficient of thermal expansion (CTE) of 11 με/°C with the hardened HSC. Besides, a thermocouple was placed horizontally in the center of the mold to evaluate the temperature of HSC (Figure 1a). The top surface of each prismatic specimen was sealed with a polyester film to avoid moisture evaporation after HSC mixtures casting. After 24 h, some of the specimens were demolded and exposed to an air environment to evaluate the drying shrinkage, in contrast, some of the specimens were demolded and kept sealed (Figure 1b).

### 3.4. Rapid Freezing and Thawing Resistance Test

Two prismatic specimens (100 × 100 × 400 mm)) of each mixture were prepared and tested under repeated freezing from 4 °C to −18 °C and thawing from −18 °C to 4 °C cycles following ASTM C666 [20]. The specimens were evaluated for their relative dynamic modulus of elasticity (*E_ft_*) and their mass loss rate (*m_ft_*) every 30 cycles until 300 freezing-thawing cycles were completed. Furthermore, the influence of freezing and thawing cycles on the HSC with EAS under static strength test (flexure, and unconfined compressive strength) is still unclear, thus, a flexure and compressive strength test was adopted after 300 cycles. The four points flexural test was performed using the freezing-thawing specimens following ASTM C1609 [21]. Next, carried out compressive strength tests using half specimens from the flexural test (100 × 100 × 100 mm).

## 4. Results and Discussion

### 4.1. The XRD Results

The aging process of EAS can significantly reduce free-CaO to make stable EAS powder, while it seems to bring about the low cementitious activity. The cementitious activity of EAS can be improved with the increase in fineness [22,23]. Mason et al. [24] proposed an equation of the basicity (CaO/SiO_2_ + P_2_O_5_)) to evaluate the hydration activity of supplementary cementitious materials, in which a value larger than 1.60 is defined as supplementary cementitious materials have cementitious properties (consist main content of C2S and C3S). The basicity of EAS and GBS exhibited 1.64 and 1.70, respectively, based on Table 2, which means the EAS can be used as supplementary cementitious materials.

**Figure 2:**
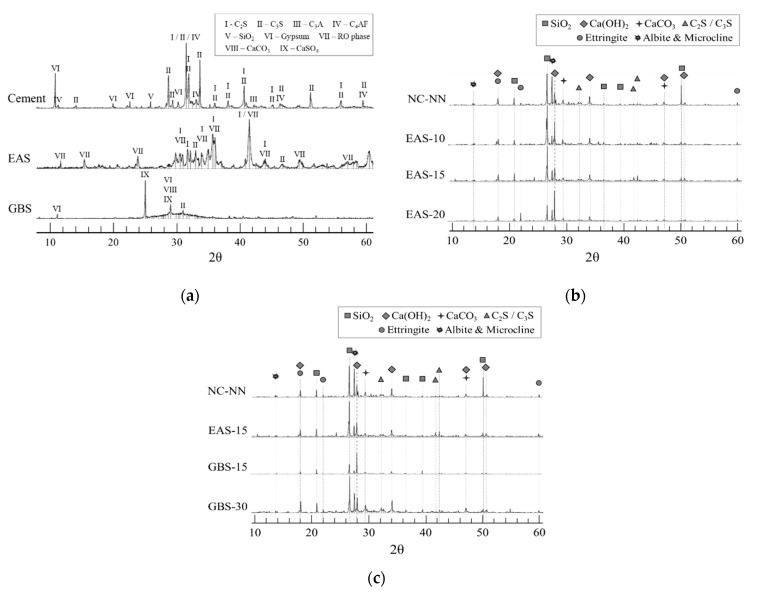
XRD analytical results: (**a**) comparison of cementitious materials; (**b**) comparison of EAS replacement ratio; (**c**) comparison of EAS and GBS.

Figure 2 shows the XRD patterns of the cementitious materials (cement, EAS, and BSF) and HSC specimens after 28 days. The crystalline phases of the cementitious materials are presented in Figure 2a. The main mineral phases C3S, C2S, C3A, and C4AF were detected in the cement. Typical minerals in EAS include C3S, C2S, and RO phase. The peak intensities of active minerals (C3S, C2S) in the EAS are significantly lower than in cement. The RO phase is well-crystallized and inert in EAS and mostly unhydrated even under strongly alkaline conditions [25].

Figure 2b,c show the XRD results of the HSC specimens with EAS and GBS at 28 days. The crystallized in the hydration products of HSC specimens with and without EAS or GBS mainly come from Ca(OH)_2_, CaCO_3_, Ettringite, and some dehydrated cement clinkers (C2S and C3S). The pozzolanic reaction was still activated after 28 days according to the peaks related to Ca(OH)_2_ continuously showing in the XRD patterns of each specimen [26]. The unreacted raw materials of tricalcium (C3S) and dicalcium (C2S) silicates are also largely crystallized in the hydration products of the specimens. The NC-NN, EAS-10, EAS-15, and GBS-15 of HSC specimens exhibited smaller quantities of C2S and C3S than other specimens. This means that NC-NN, EAS-10, EAS-15, and GBS-15 of HSC specimens have reached a more mature hydration stage [27].

### 4.2. TG/DTG Analysis

**Figure 3:**
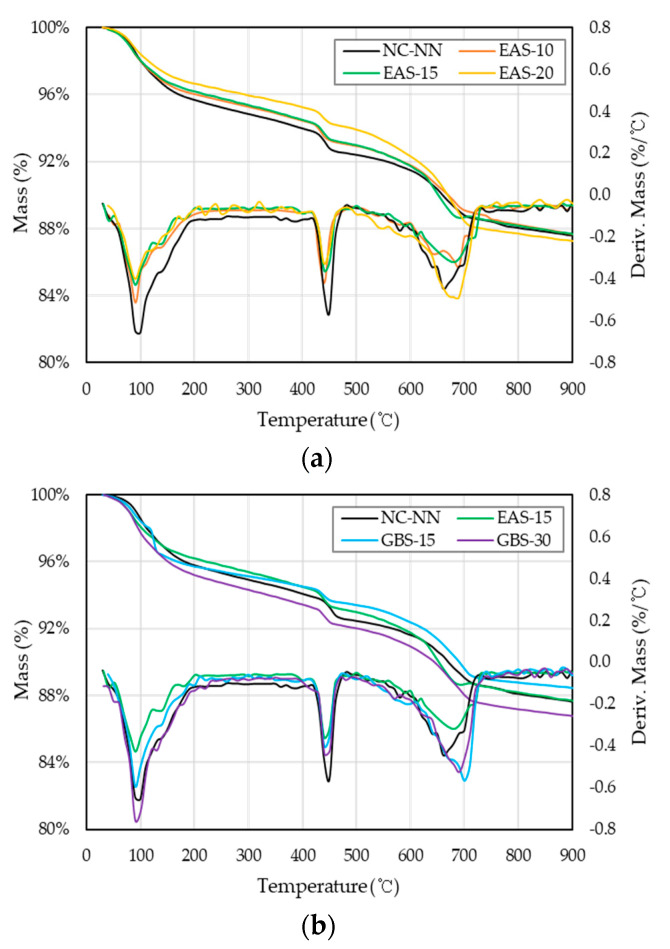
TG and DTG curves for HSC mixtures: (**a**) comparison of EAS replacement ratios; (**b**) comparison of EAS and GBS.

Thermogravimetry (TG) and Derivative Thermogravimetry (DTG) analytical methods provide the structural properties relationship with the thermal stability of the materials. This analytical method is a simple and fast way to understand some information about the stability and decomposition of a material [28,29]. The TG and DTG curves of HSC specimens are shown in Figure 3 According to the dehydration of a large proportion of Ettringate and some calcium silicate hydrate (C-S-H) gel, all HSC specimens have an endothermic peak around 100 °C. The decomposition peaks of Ca(OH)_2_ and CaO_3_ appear at approximately 450 °C and 650 °C, respectively. In the specimens of HSC with EAS, the decomposition peaks of Ca(OH)_2_ and CaO_3_ are smaller in the area and at a slightly lower temperature than the other specimens, this may indicate that a lot of Ca(OH)_2_ has poor crystallinity. The higher the content of EAS replacing cement in the HSC mixture, the more noticeable this phenomenon is, that is, EAS behavior will be worse than that of cement and GBS after thermal loads. This phenomenon is consistent with the results of XRD analysis, which indicates that HSC with EAS does not observably consume Ca(OH)_2_ during the concrete hydration. Nevertheless, the pozzolanic reaction consumes Ca(OH)_2_ and is conducive to the formation of C-S-H gel around the cementitious materials as well as enhance the interfacial transition zone of the concrete [1,29].

The calculated dehydration of Ca (OH)_2_, based on Taylore’s method, in specimen NC-NN is 13.2%. For specimens EAS-10, EAS-15, EAS-20, GBS-15, and GBS-30, the Ca (OH)_2_ is 6.7%, 5.4%, 4.7%, 9.9%, and 18.1%, respectively. The higher the EAS replacement ratio is, the lower the Ca (OH)_2_ content. The replacement ratio of GBS has precisely the opposite effect. The explanation for this has two aspects. First, the pozzolanic reaction of the SiO_2_ consumes Ca (OH)_2_ [13,30], hence the Ca (OH)_2_ decreases with increasing amounts of EAS in the mixture, these mixtures have less SiO_2_ content than cement and GBS, as shown in Table 2 (content of SiO_2_ shows GBS > cement > EAS). Another is that Ca (OH)_2_ produced by the hydration of C3S and C2S. and the total contents of C3S and C2S in the EAS mixtures is strongly less than in cement and GBS, as shown in Figure 2.

### 4.3. Pore Size Distribution

**Figure 4:**
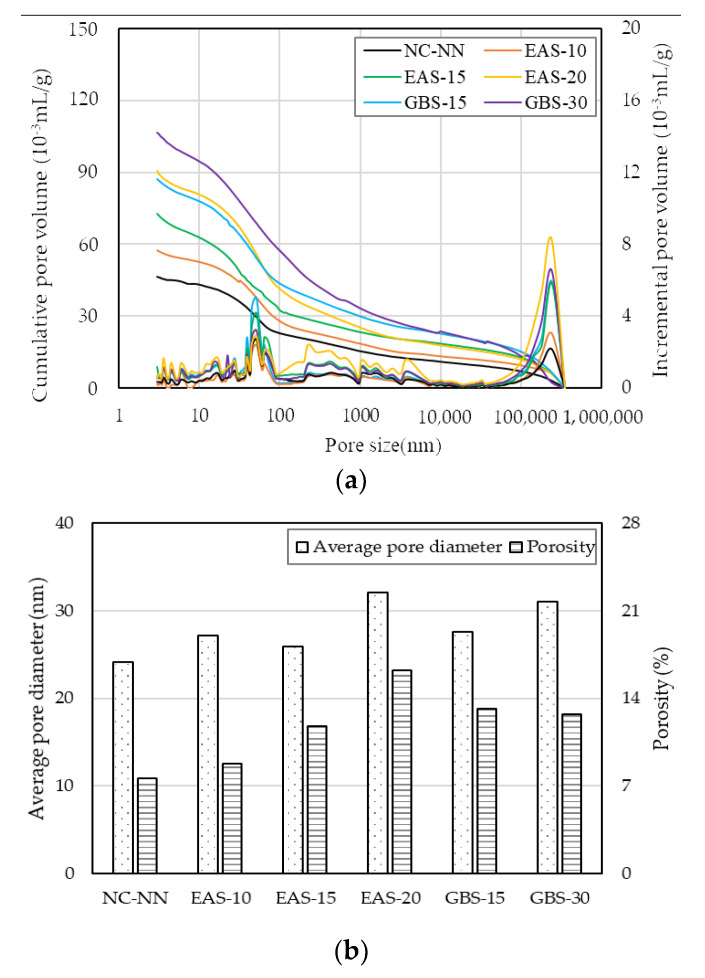
Pore size distribution of HSC at 28 days: (**a**) comparison of cementitious materials replacement ratios; (**b**) porosity and average pore size of the selected composite.

Figure 4 shows the pore volume distribution of the selected HSC specimens after 28 days. The size of the matrix pore can be used to define the pore distribution, and the pore distribution is directly proportional to the average pore [25,29,30]. The pore size distribution centralizes mainly in the range of 5–80 nm, which is closely related to the dissolution of micro cementitious materials and the formation of hydration products [27,31].

Figure 4a shows that the pore volume, related to the pore diameter, is the lowest in specimen NC-NN and increased with the increasing replacement ratio of EAS. This can be explained by the pozzolanic reaction of SiO_2_ with Ca(OH)_2_ lead to a denser concrete matrix and a more mature hydration stage as shown in the XRD analysis. The hydration products in NC-NN are greater than those in HSC with cementitious materials, resulting in a lower pore volume in the NC-NN. It could also be caused by EAS containing some free lime (*f*-CaO), which leads to deleterious expansion and volume instability [6,7,8,9]. In the HSC with GBS, the pore volume was slightly higher than in other specimens, which is presumably because of the lower surface area of GBS. Moreover, pores over 1000 nm could have a negative effect on strength development.

Furthermore, as seen in Figure 4b, the specimen NC-NN has a smaller average diameter and lower porosity of, 24.1 nm and 16.9%, respectively. The HSC with EAS specimens had 7.5–33.2% higher average pore diameter (25.9–32.1 nm) and 15.3–113.2% larger porosity (12.57–23.24%) compared to NC-NN. The HSC with GBS specimens also had a smaller average pore diameter and lower porosity, by approximately 3.4–14.0% and 19.0–21.9%, respectively, compared to the HSC with EAS. The average pore diameter and porosity are mainly influenced by the EAS and GBS replacement ratios, i.e., these properties increase with increasing replacement ratios of EAS and GBS.

### 4.4. Compressive Stregnth Properties

**Figure 5:**
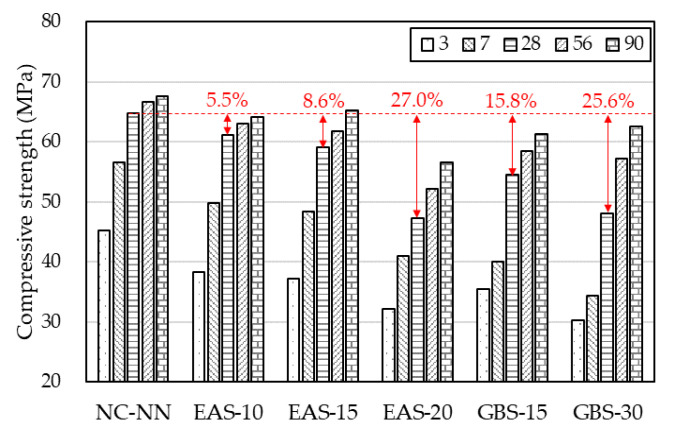
Test values for compressive strength.

Figure 5 shows the compressive strengths of the HSC at 3, 7, 28, 56, 90 days. The early compressive strength of NC-NN is significantly higher than that of HSC containing EAS or GBS. This is due to the hydration activity of EAS and GBS being substantially less than cement at an early age, EAS, in particular, has a retarding effect on the hydration activity of the cement matrix [1,10,32]. According to Figure 5, at the age of 28 days, when increasing the replacement ratio of EAS has reduced the compressive strengths of HSC, by approximately 5.5%, 8.6%, and 27.0% for EAS-10, EAS-15, and EAS-20, respectively, compared to NC-NN. The compressive strength of HSC with GBS is slightly lower than that of HSC with EAS, except for the specimen EAS-20. This indicates that specimen EAS-20 contains RO phase, which has a negative effect on adhesion and might lead to a lower strength than concrete without EAS [10]. Another reason is that EAS contains some of the free lime (*f*-CaO), which leads to deleterious expansion and volume instability [6,7,8,9]. The compressive strength of HSC with GBS is slightly lower than that of the others, which is due to the pore volume of HSC with GBS being slightly higher than the other specimens, especially with the range of its pore size being over 1000 nm, as shown in Figure 4.

**Figure 6:**
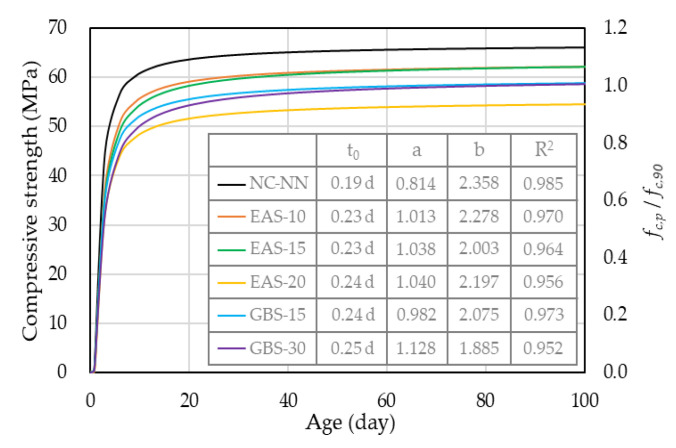
Prediction values for compressive strength.4.5. Shrinkage Behavior under Free Condition.

According to the unconfined compressive strength test results obtained at specific times (3, 7, 9, 28, 56, 90 days), an equation for predicting the compressive strength development of HSC with age is proposed. Based on previous research [33], the strength development of HSC has an almost linear relationship with the hydration degree. Therefore, Equation (1) was derived to predict the compressive strength development of HSC based on the hydration degree model [34,35] as follows:

(1) $$f_{c,p}=\;f_{c,90}\times e^{-a\;\times \;{\left[In\left(1+\left(t-t_{0}\right)\right)\right]}^{-b}}$$

where *f_c_*_,90_ is the unconfined compressive strength after 90 days, *t*_0_ is the time when the shrinkage stress first develops, and a and b are the regression coefficients (summarized in Figure 6).

Equation (1) involves the 90 days compressive strength, which is similar to Grybeal’s formula [36], and the predicted behavior is following the experimental results.

### 4.5. Autogenous Shrinkage Behavior

#### 4.5.1. The Start Time of Shrinkage

**Figure 7:**
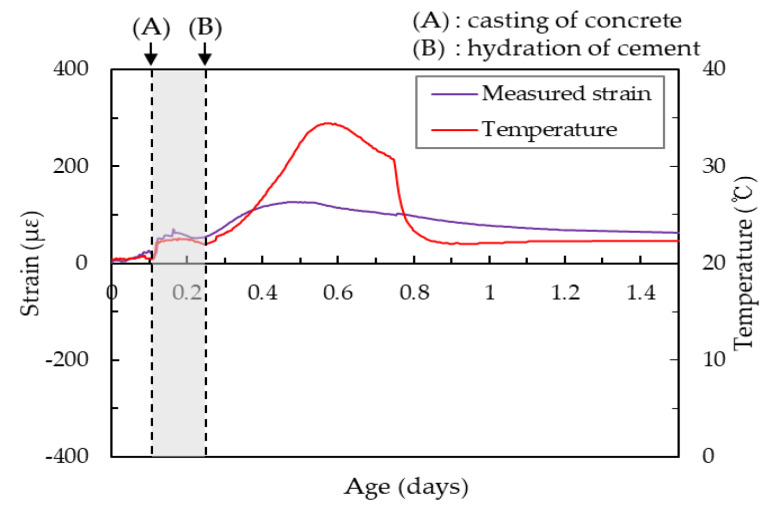
Initial strain and temperature behavior of HSC.

Figure 7 shows the initial strain and temperature behaviors of the HSC. Point (A) indicates the start time of pouring the HSC in the shrinkage mold. The strain measured by the embedded gage in the HSC immediately increased with increasing temperature (measured by a thermocouple). Due to the frictional heat during mixing, the temperature of the fresh HSC mixture was higher than the room temperature (20 °C). Then, the strain and temperature of the HSC gradually approached room temperature. This is because the variations in strain and temperature were mainly influenced by the temperature differential between the fresh HSC and the room. In this phase, the strain and temperature were not influenced by the hydration heat and shrinkage of HSC [37,38]. The temperature then started to increase according to the hydration heat of the HSC, and the HSC expanded according to the hydration heat at point (B). That is, the strain and temperature values before point (B) were not associated with the evaluation shrinkage of HSC [35,37]. Therefore, point (B) is assumed to be the start time (time-zero) for shrinkage in this study.

#### 4.5.2. Early Age Behavior of the HSC

The autogenous shrinkage of HSC under free conditions is caused by the chemical shrinkage affiliated with hydration and by moisture being transferred between the interior voids and not out to the exterior surrounding environment [19]. Thus, the top surface of HSC prismatic specimens was sealed with a polyester film to avoid moisture evaporation in this study. The thermal dilation of test specimens necessary to calibrate the strain measurement in to evaluate the pure autogenous shrinkage is as follow:

(2) $$\varepsilon_{\mathrm{as}}={\;\varepsilon }_{\mathrm{ms}}-\;\alpha \Delta T$$

where ε_ms_ is the autogenous shrinkage measured in the test, α is CTE of HSC, ∆T is the temperature variation.

**Figure 8:**
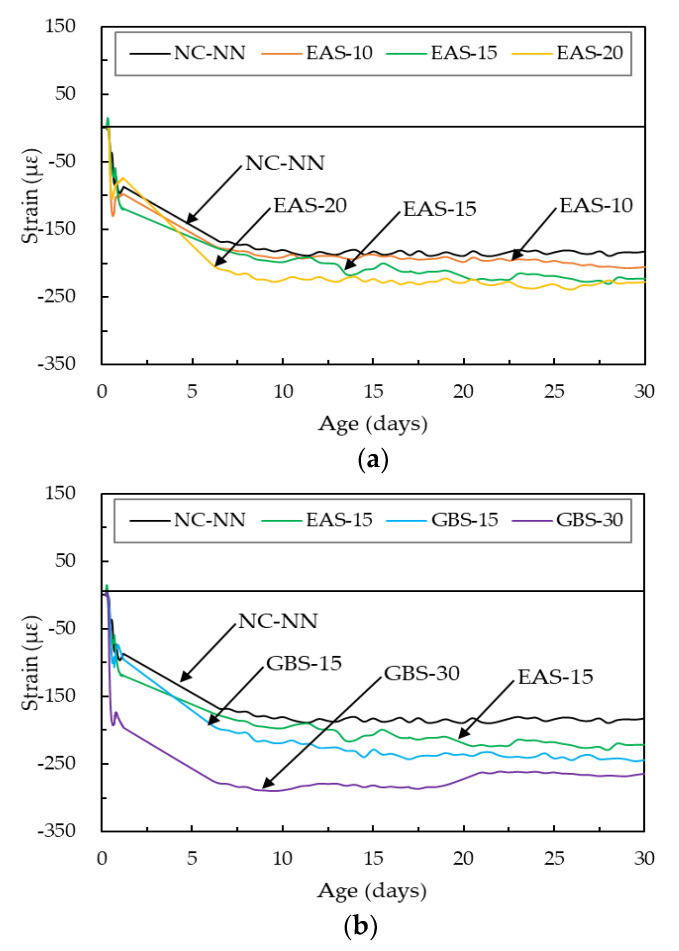
Autogenous shrinkage behavior: (**a**) HSC containing EAS; (**b**) HSC containing GBS.

Figure 8 shows the autogenous shrinkage of HSC with and without EAS and GBS. All specimens showed similar shrinkage behavior at 30 days, where autogenous shrinkage steeply increased after time-zero. The CTE of the HSC was used a value of 11 με/°C in this study, this has been suggested by previous researchers [35,37]. Some slight expansion behavior had occurred after a certain point (13.8 h for NC-NN, 14.1 h for EAS-10, 14.7 h for EAS-15, 21.0 h for EAS-20, 16.0 h for GBS-15, 20.5 h for GBS-30). This is because of the self-restrained chemical shrinkage due to the hardening of the concrete base and the volume contraction from the negative pressure in the internal voids [35,39]. The autogenous shrinkage of HSC with EAS is similar to that of NC-NN when the replacement ratio was less than 15%. However, the specimen of HSC with 20% EAS has significantly higher autogenous shrinkage than NC-NN. As for shrinkage of HSC with GBS, increases with increasing replacement ratios of GBS. This is because greater replaced cementitious material results in increased micro-pore distribution and moisture loss as shown in Figure 4.

**Figure 9:**
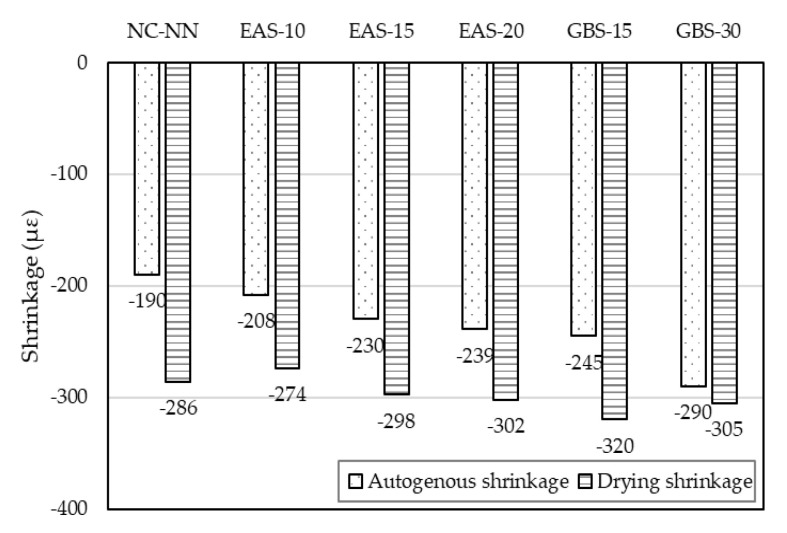
Shrinkage behavior under free conditions.

The drying shrinkage of concrete is related to the loss of moisture from the concrete [40]. Liu et al. [1] published a paper stating that drying shrinkage of concrete increases with EAS replacement ratio and the shrinkage of concrete with over 15% EAS is obviously higher than that of concrete without cementitious materials. The same phenomenon can be seen in this study. The drying shrinkage of EAS-10 and EAS-15 is similar to NC-NN, they are approximately −274 με, −298 με, and −286 με, respectively (Figure 9). The drying shrinkage of EAS-20, GBS-15, and GBS-30 are all higher than NC-NN, for the same reason as the autogenous shrinkage being higher. Overall, the free shrinkage of HSC containing the smaller than 15% of EAS is close to HSC without cementitious material.

### 4.6. Effect of Freezing and Thawing Cycles

**Figure 10:**
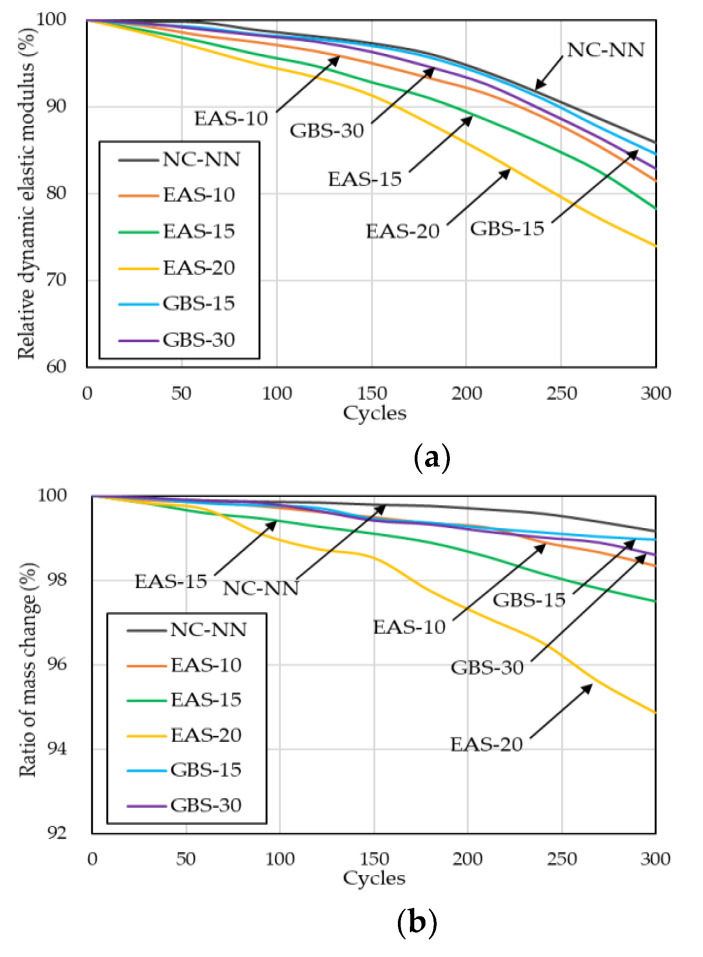
Results of rapid freezing and thawing resistance test: (**a**) relative dynamic modulus of elasticity; (**b**) ratio of mass change.

**Figure 11:**
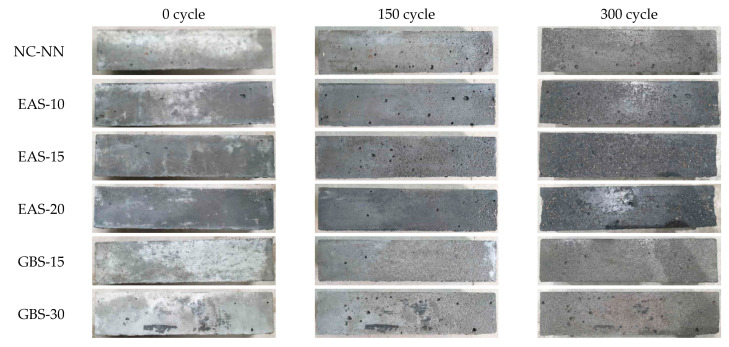
Concrete surface scaling after various amounts of freezing and thawing cycles.

Figure 10 presents the rapid freezing and thawing test results over 300 cycles. The HSC mixtures containing EAS showed a quite gradual decrease of 18.6–26.1%, and 1.66–5.13% in the *E_ft_* and m_ft_, respectively. Especially, specimen EAS-20 showed rapid deterioration after 150 cycles with *E_ft_* and m_ft_ reduced approximately to 73.9%, and 94.9%, respectively. This is because the replaced EAS results in increased micropore distribution and moisture loss as shown in Figure 4. The internal pressure of ice in the internal void of the concrete acted with thermal changes to produce superficial spalling in the specimen and leads to a rapid decrease in their *E_ft_* and m_ft_. Whereas the HSC mixtures containing GBS had similar test result values and behavior when compared with NC-NN. This result is in agreement with the report from Hooton et al. [41] and from Nakamoto et al. [42], which also states that there is no detrimental effect of GBS on the freezing and thawing resistance of concrete. Therefore, the HSC containing 20% EAS may deteriorate due to freezing-thawing action. In contrast, HSC containing less than 15% EAS may have sufficient resistance to deterioration from freezing-thawing action. Figure 11 shows three typical cycles (0, 150, 300 cycles) of HSC specimens where the surface visually confirms the mass changes of the specimen.

Table 3 shows a comparison with strength (flexure and compressive strength) before and after the freeze-thaw cycle. The internal pressure of ice in the internal void of the concrete acted with the thermal changes to produce superficial spalling in the specimen and leads to the rapid decrease in their strength. The loss rate of HSC increases with the increasing replacement ratio of slag, especially for, HSC containing EAS. In the flexure strength test, the loss rate was significant over 67% compared to before the freeze–thaw cycles in all mixtures. As above, the HSC containing 20% EAS shows a significant loss of strength. In summary, the partial replacement of cement with EAS (<15%) can improve strength and shrinkage compared to that of GBS, however, the freeze-thawing resistance should be carefully controlled.

## 5. Conclusions

This research evaluated the shrinkage as well as the strength after freezing and thawing of high-strength concrete containing electric arc oxidizing slag. A variety of experimental tests were performed to evaluate the microstructure, strength, shrinkage, and rapid freezing-thawing resistance. From these tests, the following concluding remarks were drawn:

(1) HSC with and without slag have a similar hydration process. However, HSC containing less than 15% EAS has lower quantities of C2S and C3S, indicating it has reached a more matured hydration stage compared to HSC with higher EAS content.
(2) HSC containing EAS, when compared to the hydration product of other mixtures, has lower amounts of Ca(OH)_2_ with relatively poor crystallinity. This is because in the pozzolanic reaction, SiO_2_ consumes Ca (OH)_2_, hence Ca (OH)_2_ decreases with the increasing amount of EAS, so the total contents of C3S and C2S in EAS are less than in other HSCs.
(3) The porosity and pore volume distribution is affected by increasing EAS content; these both increase with increasing replacement ratios of EAS. A similar pattern emerges in the compressive strength test results, especially for the EAS-20 specimen that has the lowest strength among all the tested HSCs. This indicates that specimen EAS-20 contains a considerable amount of RO phase, this has a negative effect on adhesion and might lead to lower strength concrete than that without EAS.
(4) In terms of shrinkage behavior under free conditions, the HSC mixtures’ shrinkage increases with increasing replacement ratios of cementitious materials. The autogenous and drying shrinkage of HSC containing EAS is similar to that of NC-NN when the replacement ratio is less than 15%. This is because replacing the cementitious materials results in increased micro-pore distribution and moisture loss.
(5) From the freezing and thawing resistance tests, the HSC mixtures containing EAS showed a quite gradual decrease in their relative dynamic modulus of elasticity and a gradual increase in their mass-loss rate. The HSC containing 20% EAS significantly deteriorated after the freezing-thawing cycles; whereas HSC containing EAS less than 15% may have sufficient resistance to deterioration from freezing-thawing action. Furthermore, in the comparison of strength before and after freeze-thaw cycling, the loss rate of HSC increases with the increasing replacement ratio of slag, especially for, HSC containing over 15% EAS.

## Figures and Tables

**Table 1:** Mixture proportions of HSC.

Specimens	w/b (%)	*s/a* (%)	Unit Weight (kg/m^3^)				EAS (%)	GBS (%)	AEWE Agent	Slump (mm)
			Water	Cement	Sand	Coarse Aggregate				
NC-NN	32.5	40.3	170	523	656	971	-	-	0.72	172
EAS-10				471			10	-	0.72	180
EAS-15				445			15	-	0.65	170
EAS-20				418			20	-	0.65	170
GBS-15				445			-	15	0.68	180
GBS-30				366			-	30	0.65	175

(Note) w/b: water to binder ratio; *s/a*: ratio between amount of fine aggregate and total amount of aggregate; EAS: electric arc furnace oxidizing slag; GBS: ground granulated blast furnace slag. AEWR agent: air-entraining and water-reducing agent.

**Table 2:** Chemical and physical properties of cementitious materials.

Type	Surface Area (cm^2^/g)	Density (g/cm^3^)	Chemical Composition (%)							
			SiO_2_	CaO	Al_2_O_3_	T-Fe*	MgO	SO_3_	MnO	P_2_O_5_
Cement	3400	3.15	22.0	64.2	5.5	3.0	1.5	2.0	-	0.89
EAS	4893	3.96	14.2	24.1	11.1	39.92	3.33	0.019	5.59	0.537
GBS	3258	2.90	29.1	49.4	12.7	0.38	5.67	0.532	0.29	0.015

(Note) T-Fe*: total Fe_2_O_3_ and FeO.

**Table 3:** Properties of HSC containing slag after freezing and thawing cycles.

Title	Flexure Strength (MPa)			Compressive Strength (Mpa)		
	Before	After	Loss Rate	Before	After	Loss Rate
NC-NN	11.7	3.7	68.2%	66.7	53.6	19.7%
EAS-10	8.5	2.1	75.0%	58.9	44.3	24.9%
EAS-15	8.7	1.6	81.5%	56.7	39.9	29.7%
EAS-20	7.7	0.8	89.7%	48.3	19.6	59.4%
GBS-15	10.1	3.2	68.4%	58.4	48.6	16.8%
GBS-30	9.3	3.1	67.2%	58.7	47.9	18.4%

## Data Availability

The data presented in this study are available on request from corresponding author.
